# NMDA Receptors Regulate the Structural Plasticity of Spines and Axonal Boutons in Hippocampal Interneurons

**DOI:** 10.3389/fncel.2017.00166

**Published:** 2017-06-12

**Authors:** Marta Perez-Rando, Esther Castillo-Gómez, Ramon Guirado, José Miguel Blasco-Ibañez, Carlos Crespo, Emilio Varea, Juan Nacher

**Affiliations:** ^1^Neurobiology Unit, Department of Cell Biology, Interdisciplinary Research Structure for Biotechnology and Biomedicine (BIOTECMED), Universitat de ValènciaValència, Spain; ^2^CIBERSAM: Spanish National Network for Research in Mental HealthMadrid, Spain; ^3^Fundación Investigación Hospital Clínico de Valencia, Instituto de Investigación Sanitaria (INCLIVA)València, Spain

**Keywords:** NMDAR, spine dynamics, interneurons, organotypic cultures, MK-801, axonal boutons

## Abstract

N-methyl-D-aspartate receptors (NMDARs) are present in both pyramidal neurons and interneurons of the hippocampus. These receptors play an important role in the adult structural plasticity of excitatory neurons, but their impact on the remodeling of interneurons is unknown. Among hippocampal interneurons, somatostatin-expressing cells located in the stratum oriens are of special interest because of their functional importance and structural characteristics: they display dendritic spines, which change density in response to different stimuli. In order to understand the role of NMDARs on the structural plasticity of these interneurons, we have injected acutely MK-801, an NMDAR antagonist, to adult mice which constitutively express enhanced green fluorescent protein (EGFP) in these cells. We have behaviorally tested the animals, confirming effects of the drug on locomotion and anxiety-related behaviors. NMDARs were expressed in the somata and dendritic spines of somatostatin-expressing interneurons. Twenty-four hours after the injection, the density of spines did not vary, but we found a significant increase in the density of their *en passant boutons (EPB)*. We have also used entorhino-hippocampal organotypic cultures to study these interneurons in real-time. There was a rapid decrease in the apparition rate of spines after MK-801 administration, which persisted for 24 h and returned to basal levels afterwards. A similar reversible decrease was detected in spine density. Our results show that both spines and axons of interneurons can undergo remodeling and highlight NMDARs as regulators of this plasticity. These results are specially relevant given the importance of all these players on hippocampal physiology and the etiopathology of certain psychiatric disorders.

## Introduction

The study of changes in the morphology of neurons is important for understanding neural activity. In fact, changes in the dendritic arbor or the number of dendritic spines and axonal *boutons*, allow neurons to modify the strength of their synaptic input and output and to adapt to changing environments (for a review see Whitt et al., 2014).

Dendritic spines are membranous protrusions, which can establish excitatory or inhibitory synapses through different neurotransmitter receptors. Although they were thought to be a distinctive feature of principal neurons, different studies have shown that some interneuronal subpopulations also display these postsynaptic specializations (Freund and Buzsáki, 1996). In interneurons, however, the structure of the spines is slightly different: they lack the spine apparatus found in pyramidal neurons, several synapses are established per spine (in contrast with the one or two found in pyramidal neuron spines) and they are usually less numerous (Gulyás et al., 1992; Acsády et al., 1998). On the other hand, *boutons* are axonal thickenings, which contain and release the synaptic vesicles. Because of their roles as postsynaptic and presynaptic elements, spines and *boutons* have been found to be proper markers for neuronal input and output; therefore, increases in spine and axonal *bouton* density have been correlated to increases in neuronal activity (Engert and Bonhoeffer, 1999; Becker et al., 2008).

Excitatory neurons experience structural remodeling under different conditions, involving changes in the length and complexity of dendritic arbors, and in the density or morphology of their spines (Fu and Zuo, 2011) and axonal *boutons* (Florence et al., 1998; Colicos et al., 2001; Nikonenko et al., 2003). This has been shown in different conditions and disorders, including chronic stress and depression (McEwen, 1999; Qiao et al., 2016), obesity (Dingess et al., 2016), neurodevelopmental disorders (Glausier and Lewis, 2013; Flores et al., 2016) and after different pharmacological manipulations (Guirado et al., 2009; Yang et al., 2015; Castillo-Gómez et al., 2016b). By contrast, studies on the effects on interneuron morphology are scarcer, despite the important role of inhibitory networks in central nervous system physiology (Nacher et al., 2013). Most of these studies on interneuron plasticity are focused on basket interneurons and few have explored structural changes on dendrite-targeting interneurons, such as those expressing somatostatin. These interneurons are essential for the maturation of deep cortical circuits (Tuncdemir et al., 2016) and are important players in other stages of neurodevelopment, brain pathology and neuronal plasticity (for a review see Liguz-Lecznar et al., 2016). Only some recent studies have shown that these interneurons are able to undergo dendritic remodeling after chronic stress (Gilabert-Juan et al., 2011), antidepressant treatment (Guirado et al., 2014b), streptozotocin-diabetic challenge (Castillo-Gómez et al., 2015) or the depletion of plasticity related molecules (Guirado et al., 2014a; Castillo-Gómez et al., 2016a).

However, most of these structural analyses, as the majority of those in other interneuronal subtypes, have been performed on fixed tissue. Data acquired with this experimental approach is based on population analysis and does not allow for the longitudinal study of individual spines or *boutons*. Using such methods, neither transient nor homeostatic changes can be detected, i.e., changes that only alter the composition of synapses and leave the total number of spines or dendritic length intact. Structurally, these changes in network configurations are represented by the addition of one spine on one site, compensated by the pruning on another. The emergence of 2-photon microscopy and the chronic implantation of cranial windows have allowed for longitudinal analysis of individual elements of the neocortex after sensory deprivation, which have shown structural remodeling of excitatory (Hofer et al., 2009; Holtmaat and Svoboda, 2009; Cane et al., 2014), and inhibitory microcircuits (Chen et al., 2011a,b, 2012; Keck et al., 2011; van Versendaal et al., 2012; Chen and Nedivi, 2013). However, this technique is limited by the position of the cranial window and can only be used chronically, without brain damage, on studies of the neocortex. This inconvenience hinders the longitudinal study of the hippocampus, an important structure interconnected with the neocortex and responsible for spatial memory. The use of entorhino-hippocampal organotypic cultures, with their intrinsic limitations, helps to solve this problem and allows us to follow elements from a hippocampal neuron throughout an entire experiment. These cultures have been broadly used as an *in vitro* model of the rodent hippocampus (Stoppini et al., 1991), allowing the study of structural changes in real-time.

N-methyl-D-aspartate receptors (NMDARs) are a subtype of ionotropic glutamate receptors, expressed widely in both pyramidal neurons and interneurons (Collingridge et al., 1983; Nyíri et al., 2003; Alvarez et al., 2007; Oren et al., 2009). They play a key role in several events of central nervous system development, such as neuronal birth and migration (Komuro and Rakic, 1993). Antagonists to these glutamate receptors, such as MK-801, are known to interfere with the targeting and pruning of axons and the regulation of synaptogenesis during development (Cline and Constantine-Paton, 1990; Shatz, 1990; Butler et al., 1998). NMDAR antagonists also induce axonal sprouting during adulthood (Sutula et al., 1996; McKinney et al., 1999b) and are able to modulate some processes related to learning, such as LTP (Bailey et al., 1996). The blockade of these receptors does not appear to produce effects on the dendritic spine density of pyramidal neurons, neither *in vivo* (Woolley and McEwen, 1994) nor *in vitro* (McKinney et al., 1999b). However, this latter study showed the apparition of filopodia-like processes after chronic treatment with MK-801, resembling those in the developing hippocampus. Interestingly, NMDAR blockade with MK-801 increases the motility of dendritic spines of pyramidal neurons in hippocampal organotypic cultures (Alvarez et al., 2007). Regarding interneurons, little is known about the expression of NMDARs in these cells or about how the hypofunction of these receptors may affect their structure or physiology. Some studies in the prefrontal cortex have shown that MK-801 administration impairs the functional maturation of perisomatic inhibitory circuits expressing parvalbumin (Thomases et al., 2013) and affects differentially the physiology of these interneurons and pyramidal neurons (Wang and Gao, 2012). However, it is still unknown how this blockade may affect dendrite-targeting interneurons, such as those expressing somatostatin in the hippocampus. There are certain cell populations located in the *stratum oriens* sharing these characteristics, which are essential for the physiology of this region (Freund and Gulyás, 1997; Müller and Remy, 2014) and are known to present dynamic dendritic spines (Guirado et al., 2014a). Two of the most studied of these populations are the oriens-lacunosum moleculare (O-LM), and the hippocampo-septal (HS), interneurons (Jinno and Kosaka, 2002; Gulyás et al., 2003). The former are named after their microcircuitry: they receive their inputs in the *stratum oriens* from pyramidal neurons of the *stratum pyramidale* and reciprocally inhibit these cells and other interneurons, establishing these synapses in the *stratum lacunosum moleculare* (Müller and Remy, 2014). These cells are essential for the correct functioning of the hippocampus. In fact, they appear to mediate *theta* oscillations (Katona et al., 2014) and have been postulated to be essential for the establishment of spatial context-fear conditioning (Lovett-Barron et al., 2014; Müller and Remy, 2014).

In the present study, we aim to understand the impact of NMDAR manipulation on the structure and dynamics of somatostatin-expressing interneurons in the CA1 *stratum oriens*. In order to achieve our goal, we have used a transgenic mice strain in which these interneurons are constitutively labeled with enhanced green fluorescent protein (EGFP), which allows us to study their entire morphology (Oliva et al., 2000). After demonstrating the presence of NMDARs on these interneurons and their dendritic spines, we have acutely treated adult mice with the NMDAR antagonist MK-801 and have studied its effects on the structural remodeling of their dendritic spines and axonal *en passant boutons* (EPB). To have a behavioral readout of the efficacy of the treatment, we have measured locomotor activity, anxiety-related behavior and working memory. Finally, in order to study whether NMDAR manipulation modified spine dynamics in these cells, we have acutely blocked these receptors with MK-801 in entorhino-hippocampal organotypic cultures and have observed morphological changes in real-time.

## Materials and Methods

### Animals and Treatment

For both *in vivo* and *in vitro* experiments we used the EGFP-expressing inhibitory neurons (GIN), Tg(GadGFP)45704Swn mice (Jackson laboratories, Bar Harbor, ME, USA). They constitutively express the EGFP in a subpopulation of somatostatin expressing interneurons (Oliva et al., 2000).

**Figure 1:**
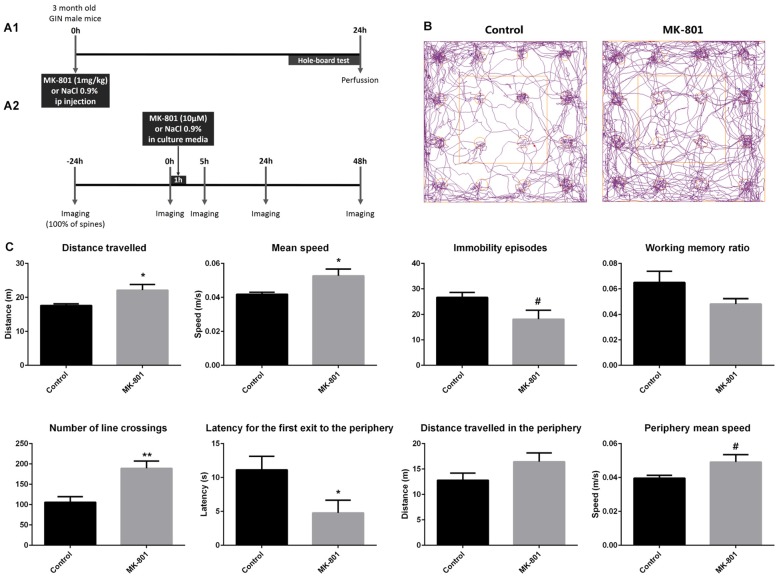
**(A1)** Experimental design of the *in vivo* experiment. **(A2)** Experimental design of the real-time *in vitro* experiment. **(B,C)** Hole-board behavioral test. **(B)** Representative tracking-plots of NaCl-treated animals (control) and MK-801-treated animals (MK-801). **(C)** Graphs representing the changes observed in the behavioral parameters that were analyzed (all graphs represent mean + SEM., *p < 0.05, **p < 0.01, ^#^0.05 < *p* < 0.1).

For the experiment analyzing *in vivo* fixed tissue, we used 3-month-old male mice (*n* = 18). The MK-801 treatment (1 mg/kg) consisted of a single intraperitoneal injection and the perfusion was performed 24 h afterwards (Figure 1A1).

For the real-time analysis *in vitro*, 14 GIN pups (P7) were used to prepare the hippocampal-entorhinal organotypic cultures (Stoppini et al., 1991). MK-801 (10 μM in culture media) was administered after the second imaging session during 1 h (Figure 1A2).

All the animals were maintained in standard conditions of light (12 h cycles) and temperature, without restrictions of food and water. All animal experimentation was conducted in accordance with the Directive 2010/63/EU of the European Parliament and of the Council of 22 September 2010 on the protection of animals used for scientific purposes and was approved by the Committee on Bioethics of the Universitat de València. Every effort was made to minimize the number of animals used and their suffering.

### Behavioral Analysis

Prior to their sacrifice, every adult GIN mouse was tested in the hole-board apparatus (ANY-maze video tracking system v4.98; Stoelting Europe, Ireland) to automatically analyze locomotor activity and anxiety-related behavior (Castilla-Ortega et al., 2010; Torres-García et al., 2012).The hole-board test measures exploration and can also be used as an initial basic screen for working memory (Karl et al., 2008). The open-field chamber (40 × 40 cm) was fitted with a hole-board floor insert for mice (16 holes, diameter = 2.8 cm). Testing of the mice took place between 1 h and 2 h after the onset of the dark phase (illumination at floor level <2 lx). Each mouse was placed in the center of the arena and was left to explore the environment for 7 min (test session). The video tracking system and the infrared photobeams provided automated measures of the: (a) total distance traveled, mean speed and immobility episodes (to study locomotor activity); (b) head dips, for the study of working memory (working memory ratio = number of head dips into novel holes/total number of head dips; Karl et al., 2008); and (c) number of entries, latency for the first entry, distance traveled and mean speed in the periphery of the arena, for the measure of anxiety and thigmotaxis (a valid index of anxiety in mice; Simon et al., 1994). The periphery zone of the area was defined as the area located between 0 cm and 10 cm from the walls of the apparatus.

### Histological Procedures

For the fixed tissue experiment, adult mice were perfused transcardially under deep pentobarbital anesthesia, with NaCl 0.9% and then 4% paraformaldehyde in phosphate buffer 0.1 M, pH 7.4 (PB). Brains were then cut with a vibratome (Leica VT 1000E, Germany) in 100 μm-thick coronal sections for the study of interneuron morphology.

Nine additional adult GIN mice were transcardially perfused for the analyses of protein expression in EGFP+ interneurons (GluN1 subunit of NMDAR), or the identification of HS cells (retrograde tracing from the medial septal nucleus). The brains were then extracted and cut in 50 μm thick coronal sections to perform immunohistochemical analyses.

### Analysis of Hippocampo-Septal Interneurons

In order to know whether some of the EGFP+ interneurons of the *stratum oriens* were projecting HS cells, we performed intracranial injections of a retrograde tracer in the medial septal nucleus of three GIN mice. Mice were anesthetized with a mixture of ketamine (50 mg/kg; Imalgene, Merial) and medetomidine (1 mg/kg; Sedator, Dechra). Carprofen (5 mg/kg; Rimadyl, Pfizer) was also injected intraperitoneally to avoid inflammation and a subcutaneous injection of butorfanol (5 mg/kg; Torbugesic, Pfizer) was administered to avoid any suffering during and after the surgery. The animals were then injected with 2% Fluorogold (Molecular Probes, Thermo Fisher Scientific, Inc., Waltham, MA, USA) in the right medial septal nucleus using a stereotaxic apparatus and borosilicate glass capillaries (World Precision Instruments, Sarasota, FL, USA; tip length >10 mm, tip thickness <50 μm). The coordinates for the injection were 0.86 mm anterior from Bregma, 0.05 mm lateral from the midline, and a 3.0, 3.5 and 4.1 mm below the pial surface, according to the adult mouse atlas (Paxinos and Franklin, 2013). After 3 days, all the animals were transcardially perfused and the brains processed for histology.

In order to image the EGFP + HS interneurons in the *stratum oriens* of CA1, we used a laser scanning confocal microscope (Olympus FV10, Japan) with a 10× objective and a 2× digital zoom. We counted 100 EGFP+ interneurons and calculated the ratio of colocalization with fluorogold.

### Analysis of GluN1 Expression

Sections from three non-treated animals were processed free-floating for fluorescence immunohistochemistry against the GluN1 subunit of the NMDA receptor. The fluorescence immunohistochemistry protocol was slightly modified in order to unmask the epitope. First, the sections were incubated at 70°C for 1 h in 0.1 M PBS and then incubated in 2 M HCl for 30 min at room temperature, as described previously by our laboratory (Nácher et al., 2007). The protocol followed as previously explained. Sections were incubated for 48 h at 4°C with rabbit anti-GluN1 (1:100, Millipore, USA). After rinsing with PBS, sections were incubated for 2 h at room temperature with donkey anti-rabbit A647 (1:400, Abcam, UK). Finally, sections were rinsed in PB 0.1 M, mounted on slides and coverslipped using fluorescence mounting medium (Dako, Germany). NDS, primary and secondary antibodies were diluted in PBS—0.2% Triton-X100.

Images were taken with a laser scanning confocal microscope using a 63× oil immersion objective with an additional 5× digital zoom for the analysis of GluN1 expression in somata and dendritic spines. Controls were performed omitting the anti-GluN1 antibody or incubating with this antibody pre-absorbed overnight with an excess of its immuno- genic peptide (LQNQKDTVLPRRAIEREEGQLQLCSRHRES, Chemicon). No immunolabeling was observed in either control.

### Analysis of the Density of Dendritic Spines and Axonal *En Passant Boutons*

The structural parameters of the GAD-EGFP+ interneurons were studied using a laser scanning confocal microscope (Leica TCS SPE, Germany), as described before (Gomez-Climent et al., 2011). Since our targets were the spiny interneurons located in the *stratum oriens*, we analyzed the dendrites arising from somata located in this *stratum* and the plexus of EGFP+ axons present in the *stratum* *lacunosum-moleculare* of the CA1 region.

For the spine density analysis, a 63× oil immersion objective and 3.5× digital zoom was used to image the different dendritic sections. Dendrites from EGFP interneurons were chosen at random, but they had to meet different criteria to be included in the study: (1) they should measure at least 150 or 200 μm from the soma; and (2) no other dendrites should be found crossing their trajectory. The data were expressed as the total number of spines in the proximal (0–50 μm), medial (50–100 μm) and distal (100–150 μm) segments from the soma. A total of six neurons were analyzed per animal.

For the density of EPB, we used a 63× oil immersion objective with a 2.5× digital zoom. Six axonal segments, measuring at least 10 μm, were chosen randomly per animal. In order to rigorously score the EPB and to avoid an overestimation, the axonal varicosities were only considered when they fulfilled three criteria: (1) they should be at least two times brighter than the axonal backbone; (2) they should be two times wider than the axonal backbone; and (3) they should not have any crossings from other axons nearby.

In order to analyze the functionality of EGFP + EPB, we studied the presence of gephyrin-expressing puncta (GEPH) in juxtaposition with these presynaptic structures. We performed a double fluorescence immunohistochemistry against gephyrin and EGFP in all the experimental animals. The immunohistochemical protocol is similar to those described above. In order to label gephyrin and to enhance the EGFP signal, we incubated all the sections with chicken anti-GFP (1:2000, Abcam, UK) and mouse anti-gephyrin (1:700, Synaptic Systems, Germany) primary antibodies for 48 h at 4^o^C. After rinsing with PBS, the sections were incubated with donkey anti-chicken CF488 (1:1000, Sigma, Germany) and donkey anti-mouse IgG A555 (1:400, Thermo Fisher Scientific, UK) for 2 h at room temperature. Sections were then rinsed with PB 0.1 M, mounted on slides and coverslipped using fluorescence mounting medium (Dako, Germany). Sera, primary and secondary antibodies were diluted in PBS—0.2% Triton-X100. All the images were taken with a laser scanning confocal microscope (Leica TCS SPE, Germany), with a 63× oil immersion objective and a 2× additional digital zoom. Six axons in the *stratum lacunosum moleculare* were analyzed per animal, following the same criteria described above for axon selection and EPB scoring. Only GEPH in juxtaposition with the EPB were counted, in order to calculate the ratio of EPB containing this synaptic marker.

### Organotypic Cultures and Real-Time Analysis

Hippocampal slices (400 μm-thick) were obtained with a tissue chopper, following the protocol described by Stoppini et al. (1991). The brain was freshly extracted from P7 mice and the hippocampus dissected along with the entorhinal cortex in order to preserve the perforant pathway. Media was changed three times per week and the cultures remained 13 days *in vitro* (13DIV) until the confocal imaging started.

For the real-time analysis of organotypic cultures, short imaging sessions (10–15 min) were carried out with a 40× water immersion objective. An additional 10× digital zoom was used to analyze dendritic segments of about 35 μm in length, located between 100 μm and 150 μm from the soma (*Z* step size of 0.8 μm). Laser intensity was kept at the minimum allowing observation, and acquisition conditions maintained unchanged over the different days of observation. Control experiments showed that this procedure did not produce any deleterious effect on cell viability, such as cell death or dendritic beadings. One dendrite was analyzed per organotypic culture slice, and 16 slices from six different animals were analyzed. All the somata of the interneurons analyzed were located in the *stratum* *oriens* of CA1 region. The imaging took place at five different time points referenced to the beginning of the treatment: −24 h, 0 h, 5 h, 24 h and 48 h, starting on DIV 13. MK-801 was added to the media after the second imaging session (0 h) for 1 h, and then again substituted by normal culture media. The rapid effect was registered after 4 h (5 h), 24 h (24 h) and 48 h (48 h).

### Statistics

After checking the normality and homoscedasticity of the data, we used a *t*-test to compare control and experimental groups. When comparing the morphological analysis in adult animals, we used an unpaired *t*-test. In the real-time longitudinal analysis, when comparing the same neuron at different time points, we used a paired *t*-test, whereas when comparing the external control (cultures receiving vehicle) to the experimental slices we used an unpaired *t*-test. In every case α was set to 0.05 and the animal or the slice was considered as the “n”.

## Results

### Increased Locomotion and Anxiety-Related Behavior on MK-801 Treated Animals

Twenty-four hours after MK-801 treatment (1 mg/kg, one i.p injection), mice showed significant alterations in locomotor activity and anxiety-related behavior when tested in the hole-board apparatus (Figures 1B,C): increased distance traveled (unpaired *t*-test; *t*_(12)_ = −2.515, *p* = 0.040), increased mean speed (unpaired *t*-tests; *t*_(12)_ = −2.600, *p* = 0.035), increased number of line crossings (unpaired *t*-test; *t*_(12)_ = −3.624, *p* = 0.003), and a decreased latency to the first exit from the center zone to the periphery (unpaired *t*-test; *t*_(12)_ = 2.321, *p* = 0.043). A trend towards an increase in the mean speed in the periphery (unpaired *t*-test; *t*_(12)_ = −2.027, *p* = 0.079) and a trend towards a decrease in the number of immobility episodes (unpaired *t*-test; *t*_(12)_ = 2.129, *p* = 0.061) could also be observed. However, working memory was not affected by the treatment (unpaired *t*-test; *t*_(12)_ = 1.739, *p* = 0.118; Figure 1C).

### Some Somatostatin Expressing Interneurons in the *Stratum Oriens* Project to the Medial Septal Nucleus

**Figure 2:**
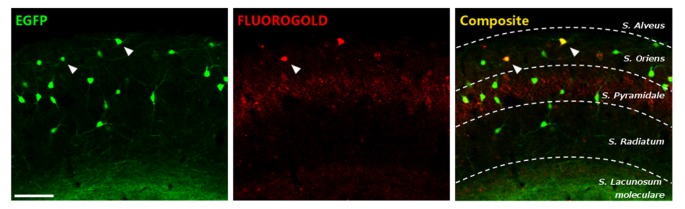
Characterization of enhanced greenfluorescent protein (EGFP)-expressing interneurons in the *stratum oriens* of CA1. EGFP+ cells (left), fluorogold retrograde tracing from the medial septal nucleus (middle) and the composite image. Scale bar: 15 μm.

In order to know the proportion of HS cells in the *stratum oriens* of GIN mice, a retrograde tracer was injected in the medial septal nucleus. This assay revealed that at least a 6.5% of EGFP+ cells were projecting to this region (Figure 2). These results indicate that, although the majority of EGFP+ interneurons in the stratum oriens of the CA1 region of GIN mice can be considered O-LM cells, some HS have also their somata among these fluorescent interneurons.

### Somatostatin Interneurons Coexpress NMDA Receptors on their Somata and on the Head of their Dendritic Spines

**Figure 3:**
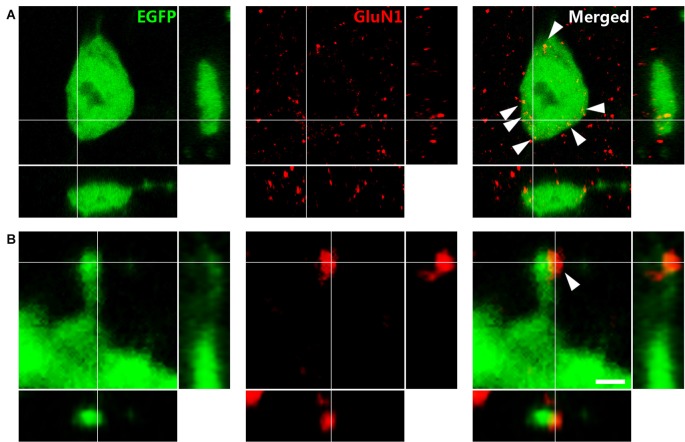
Immunohistochemistry against the N-methyl-D-aspartate receptor (NMDAR) obligatory subunit GluN1 in GIN mice.** (A)** Puncta expressing NMDARs (arrowheads) in an EGFP labeled soma. **(B)** Dendritic spine of an EFGP-labeled interneuron. Note the expression of NMDAR on the spine head. Panels on the right and bottom are orthogonal projections of the middle panel. Scale bar: 2 μm in **(A)**, 0.50 μm in **(B)**.

Immunohistochemistry against GluN1, the obligatory subunit of the NMDAR, showed wide expression in somatostatin-expressing interneurons throughout the hippocampus, as previously described (Nyíri et al., 2003). Specifically, in the somatostatin-expressing interneurons of the *stratum oriens* it was expressed on the surface of their somata (Figure 3A). Immunoreactive puncta were also found associated to the head of their dendritic spines (Figure 3B). Only one punctum, when present, appeared per spine.

### MK-801 Treatment Increases the Density of *En Passant Boutons* from Hippocampal Somatostatin-Expressing Interneurons, whereas the Density of their Dendritic Spines Remains Unaltered

**Figure 4:**
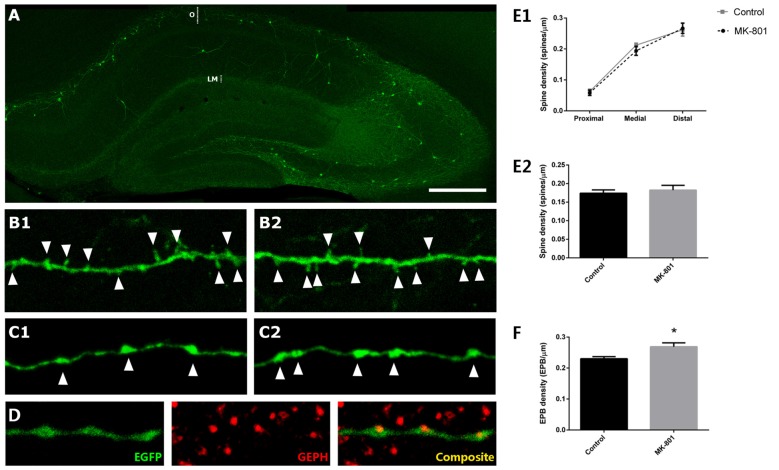
Structural modifications in EGFP-expressing interneurons in the *stratum oriens* after MK-801 injection.** (A)** Hippocampus of a GIN mouse. The *strata* *oriens* (O) and *lacunosum moleculare* (LM) are indicated with dotted lines. **(B)** Segments of dendrites bearing spines (arrowheads) from a control animal **(B1)** and a MK-801-treated mouse **(B2). (C)** Segments of axons of somatostatin-expressing interneurons in the *stratum* *lacunosum moleculare* showing *en passant* *boutons* (EPBs, arrowheads) in control **(C1)** and MK-801-treated **(C2)** animals. **(D)** Axonal segment with three *EPB* in juxtaposition to gephyrin-expressing puncta (GEPH). **(E1)** Graph representing the spine density in the proximal, medial and distal dendritic segment relative to the soma. **(E2)** Graph representing the spine density in the total length of the dendrite. **(F)** Graph showing the density of EPBs in *stratum lacunosum moleculare*, where a significant increase can be observed in the MK-801 group (all graphs represent mean + SEM., *p < 0.05). Scale bar: 180 μm in **(A)**, 7 μm in **(B)**, 5 μm in **(C)** and 7 μm in **(D)**.

We studied the densities of dendritic spines and *EPB* of somatostatin-expressing interneurons in the *stratum oriens* of CA1 (Figure 4A), 1 day after an intraperitoneal injection of the MK-801. When analyzing the different segments of the dendrite (proximal, medial and distal from the soma), we did not observe any significant difference in dendritic spine density between control and MK-801-treated animals (unpaired *t*-tests; proximal: *t*_(8)_ = −0.338, *p* = 0.744; medial: *t*_(6)_ = −0.736, *p* = 0.483; distal: *t*_(8)_ = −0.015, *p* = 0.988; Figures 4B1,B2,E1). Likewise, there were no differences between groups when comparing the total density of dendritic spines throughout the dendrite (unpaired *t*-test; *t*_(8)_ = −0.531, *p* = 0.610; Figure 3E2). However, in the *stratum* *lacunosum moleculare*, the axonal projection field of O-LM interneurons, the linear density of *EPB* was significantly increased in MK-801-treated animals (unpaired *t-test;* *t*_(8)_ = −2.220, *p* = 0.046; Figures 4C1,C2,F). Interestingly, the ratio of GEPH in juxtaposition with EPB did not vary between groups. Every EPB analyzed had an associated GEPH (Figure 4D).

### Real-Time Analysis of the Dendritic Spines of EGFP-Expressing Interneurons Reveals a Rapid Decrease on the Appearance Rate and the Dynamic Events after MK-801 Administration

**Figure 5:**
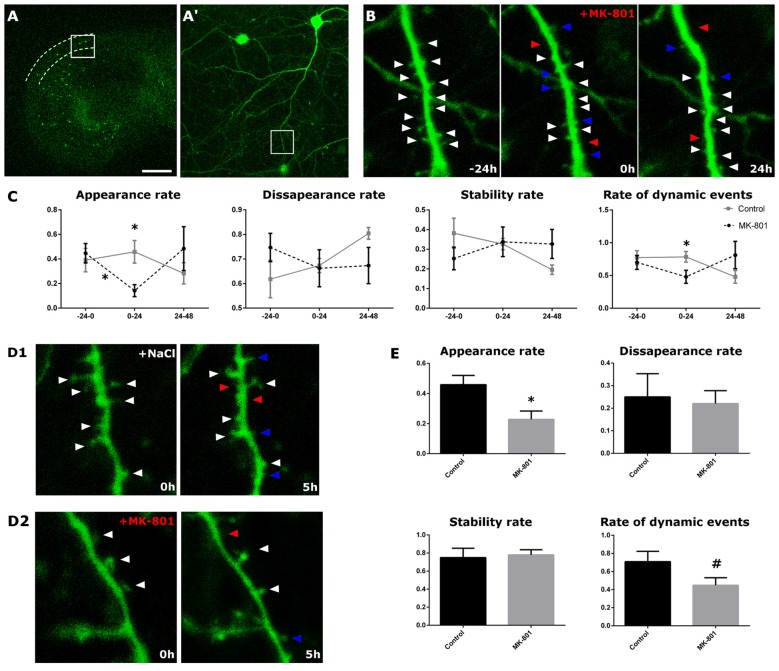
Structural dynamics of EGFP-expressing interneurons after MK-801 administration.** (A)** Panoramic view of an organotypic entorhino-hippocampal culture, with the *stratum oriens* of CA1 delineated by dotted lines. **(A′)** Enlarged view of the squared region with an interneuron in the *stratum* *oriens*. **(B)** Enlarged view of the squared region in **(A,A′)**. The figure shows a dendritic segment 24 h prior (left panel), right before (middle panel) and 24 h after (right panel) the MK-801 administration. Arrowheads point to stable (white), gained (blue) and lost (red) spines. **(C)** Graphs showing different dynamic rates in control and MK-801 treated slices (appearance, disappearance, stability rate and rate of total dynamic events). **(D1,D2)** Microphotographs showing the differences in the spine appearance rate between a control slice **(D1)** and the MK-801 treated slice **(D2)** 5 h after the starting of the MK-801 administration. **(E)** Graphs showing the different rates 5 h after the beginning of the treatment (all graphs represent mean + SEM., *p < 0.05, ^#^0.05 < *p* < 0.1). Scale bar: 1500 μm in **(A)**, 150 μm in **(A′)**, 1.5 μm in **(B,D)**.

Real-time analysis was performed in control conditions and after the acute addition of MK-801 (1 h in the culture medium; Figures 5A,A′). In control conditions, we found no alterations in the appearance, the disappearance and the stability rates, or in the rate of dynamic events. However, 24 h after the infusion of MK-801 (0–24 h), the appearance rate of new spines was significantly reduced from 46% under control conditions to a 14% of the total of pre-existing spines in treated cultures. This decrease in the appearance rate was significant when compared to both the baseline (−24 h to 0 h, paired *t*-test, *t*_(5)_ = 3.492, *p* = 0.017) and the external control (unpaired *t*-test, *t*_(8)_ = 3.338, *p* = 0.010, Figures 5B,C). The stability and disappearance rates did not change (unpaired *t*-tests; stability rate: unpaired *t*-test *t*_(8)_ = 0.119, *p* = 0.908; disappearance rate: unpaired *t*-test, *t*_(8)_ = −0.119; Figures 5B,C). In addition, there was a trend towards a decrease in the rate of dynamic events 24 h after the administration of MK-801 (unpaired *t*-test, *t*_(8)_ = 2.189, *p* = 0.060; Figures 5B,C).

We also imaged the dendrites 4 h after MK-801 administration (0–5 h), in order to register putative rapid effects of this compound. In the MK-801 group, we recorded an acute decrease in the spine apparition rate (unpaired *t*-test, *t*_(8)_ = 2.713, *p* = 0.027, Figures 5D1,D2,E). Neither the disappearance nor the stability rates varied 4 h after the treatment (unpaired *t*-tests; stability rate: *t*_(8)_ = −0.273, *p* = 0.792; disappearance rate: *t*_(8)_ = 0.273, *p* = 0.792; Figures 5D1,D2,E). However, there was a trend towards a decrease in the rate of dynamic events in the MK-801 group (unpaired *t*-test, *t*_(8)_ = 1.894, *p* = 0.095, Figures 5D1,D2,E).

### Real-Time Analysis of the Dendritic Spines of EGFP-Expressing Interneurons Shows a Decrease on the Relative Spine Density after MK-801 Administration

**Figure 6:**
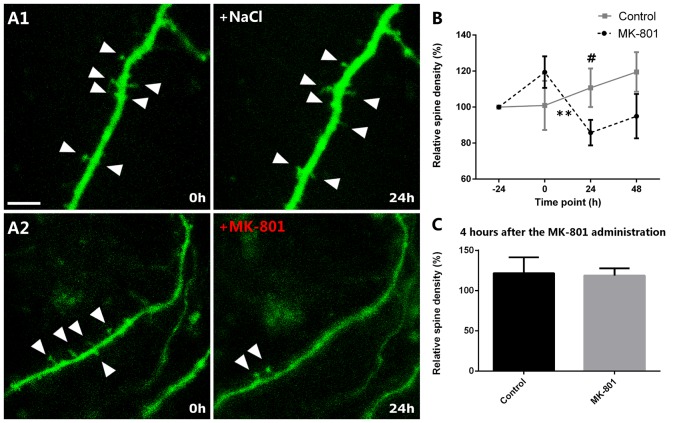
Relative spine density after MK-801 administration. **(A)** Representative images of dendritic segments in control **(A1)** and MK-801-treated **(A2)** groups. Note the decreased relative spine density 24 h after the MK-801 treatment (**A2**, right panel). Triangles mark the dendritic spines. **(B)** Graph showing the relative spine densities in the different time points. **(C)** Graph representing the relative spine density 5 h after the beginning of the MK-801 administration. Scale bar: 6 μm (All graphs represent mean + SEM., **p < 0.01, ^#^0.05 < *p* < 0.1).

When analyzing the relative variation of the spine density in organotypic cultures, we observed a marked decrease 24 h after the administration of MK-801 when compared to its group baseline. Just before MK-801 administration, the dendrites showed 119% of the original density of spines and statistically did not differ from the control group, which showed a 101% (unpaired *t*-test, *t*_(8)_ = −1.202, *p* = 0.264, Figures 6A1,A2,B). Nevertheless, 24 h after the treatment, the relative spine density was decreased to 86% in the MK-801-treated group (paired *t-test*, *t*_(5)_ = 4.827, *p* = 0.005, Figures 6A1,A2,B), and there was a trend towards a decrease when compared to the control group in the same time point (unpaired *t*-test, *t*_(8)_ = 2.035, *p* = 0.076; Figures 6A1,A2,B).

In order to analyze the acute effect of MK-801, we also studied the relative spine density 4 h after the MK-801 administration. There were no significant differences when compared to the control group (unpaired *t*-test, *t*_(8)_ = 0.150, *p* = 0.884; Figure 6C).

## Discussion

The NMDARs are among the most studied receptors in the nervous system, because of their involvement in LTP and several developmental processes (Butler et al., 1998; McKinney et al., 1999b; Nacher and McEwen, 2006; Kehoe et al., 2014). These receptors also play an important role in the activity-dependent regulation of the morphology of the dendritic spines of excitatory neurons (Nikonenko et al., 2002; Ultanir et al., 2007). However, despite the abundant presence of NMDARs in interneurons (Nyíri et al., 2003), little is known about their role on the structural plasticity of these cells. Interneurons also change their axonal and dendritic morphology in response to different stimuli (Chen et al., 2011b; Gilabert-Juan et al., 2011; Nacher et al., 2013). Furthermore, some interneuron populations have spines in their dendrites and they are dynamic, as the ones present on pyramidal neurons (Keck et al., 2011; Guirado et al., 2014a). Only some studies have focused on the structural plasticity of interneurons. Dr. Nedivi’s laboratory, using cranial windows in transgenic mice with fluorescent interneurons, has shown the elongation/retraction of dendritic branch tips *in vivo* in different neocortical regions (for a review see Chen and Nedivi, 2013). There are scarce published studies on the turnover of interneuronal dendritic spines. In mice, these spines have a stability rate close to 98% in a 24 h time-lapse experiment (Keck et al., 2011). These results are considerable higher than the ones we describe in hippocampal interneurons in the control group, where the stability rate is around 70%. These differences may be due to a difference in the stability of different interneuronal subpopulations, the region and age studied, or, more likely, to a higher stability *in vivo* than in our organotypic cultures.

The use of NMDAR antagonists, such as MK-801 or ketamine, has increased exponentially our knowledge on the physiology of these receptors and their involvement in neurological and psychiatric disorders. They have been broadly used to model certain symptoms of schizophrenia (Gilmour et al., 2012), to produce neurotoxic insults (Beninger et al., 2002) or as a novel treatment for depression (Browne and Lucki, 2013; Yang et al., 2015). In order to obtain a behavioral readout of the effectiveness of NMDAR blockade induced by *in vivo* MK-801 administration, we used the hole-board apparatus and automatically tracked the mice to test for hyperlocomotion, anxiety-related behaviors, and effects on working memory. Our study agrees with the previously reported increase in locomotor activity in MK-801 treated mice (Zuo et al., 2006; Kalinichev et al., 2008). The working memory ratio showed a non-significant decrease in the MK-801-treated animals, which is similar to the absence of differences in head-dip counts reported by other labs (Haj-Mirzaian et al., 2015; Hirose et al., 2016).

There is some evidence of the impact of NMDAR antagonists on interneurons, particularly on those targeting the soma of pyramidal neurons (Romón et al., 2011; Rotaru et al., 2011). However, little is known about the effects that NMDAR blockade can exert on other types of interneurons, like those targeting the distal segments of the dendrites of pyramidal neurons. One of the best studied populations of these dendrite-targeting interneurons are the O-LM cells in the hippocampus. These interneurons are a specially interesting cell type to study structural plasticity, because they have dendritic spines (Sik et al., 1995; Katona et al., 1999; Guirado et al., 2014a), which mainly receive excitatory synapses from recurrent collaterals of local pyramidal cells (Blasco-Ibáñez and Freund, 1995). They are essential to the proper functioning of this region of the limbic system, since they appear to modulate CA3 and entorhinal inputs (Leão et al., 2012), mediate *theta* rhythm (Katona et al., 2014) and have been associated with spatial context-fear conditioning behavior (for a review see Müller and Remy, 2014). In addition to these O-LM cells, our study, using retrograde tracing has shown that a minority of the EGFP+ interneurons in the *stratum oriens* are HS cells, suggesting that NMDA receptors may also modulate the structure of these projection interneurons.

Interestingly, different studies have found that the dendritic complexity of somatostatin-expressing interneurons can change after a chronic stress paradigm (Gilabert-Juan et al., 2017) and that they particularly change their dendritic spine density after the chronic administration of fluoxetine (Guirado et al., 2014b).

Somatostatin-expressing interneurons in the *stratum oriens* express NMDARs in their somata and dendrites (Nyíri et al., 2003). In the present study, we increase this knowledge by showing NMDAR expression specifically in the dendritic spines of these interneurons. In hippocampal pyramidal neurons, *in vitro* studies have shown that NMDARs mediate spinogenesis during neurodevelopment (Kwon and Sabatini, 2011) and are responsible for spine structural remodeling (Matsuzaki et al., 2004; Lai and Ip, 2013). Therefore, it is not unreasonable to think that NMDARs could have a similar role in interneuronal spines and mediate these types of plasticity.

To know whether NMDARs could modulate interneuronal spine density in somatostatin-expressing interneurons of the hippocampus, we have acutely injected MK-801 to adult animals. Pyramidal neurons in the mPFC modify the density of their dendritic spines after NMDAR blockade with several drugs. Previous studies have shown that after NMDAR blockade with MK-801 *in vivo*, pyramidal spine density in the hippocampus remained unaltered (Woolley and McEwen, 1994; Han et al., 2013). In accordance with these results in excitatory neurons, we have found that the acute administration of MK-801 does not affect the density of dendritic spines of somatostatin-expressing interneurons in adult mice. However, other studies with NMDAR antagonists in pyramidal neurons have rendered positive results: studies using chronic treatments have described increases (Flores et al., 2007) or decreases (Velázquez-Zamora et al., 2011) in this parameter, and those using acute administration reported a rapid increase in spine density (Li et al., 2010; Liu et al., 2013; Phoumthipphavong et al., 2016). Discrepancies in the doses and duration of the treatment may account for these differential effects, as well as differences in the structure and physiology between the spines of pyramidal neurons and interneurons (Gulyás et al., 1992; Freund and Buzsáki, 1996; Acsády et al., 1998).

There is an apparent discrepancy between the results we obtained *in vivo* and those we found in organotypic cultures. This could be due to a higher spine stability in adult animals than in organotypic cultures (Guirado et al., 2014a). Moreover, the different results may be the consequence of a different subunit composition of their NMDARs. These receptors vary their composition during the second postnatal week, when there is a developmental switch from the subunit GluN2B to the subunit GluN2A, which have different kinetic traits (Erreger et al., 2005). Our hippocampal slices have been generated and studied during this time period, when their NMDAR still have the GluN2B subunit. The treatment of organotypic cultures with MK-801 induced significant changes in the dynamics and density of dendritic spines of somatostatin-expressing interneurons. Most of these cells are probably O-LM interneurons, since the type of organotypic culture used in our study (Stoppini et al., 1991) only preserves the perforant path and the local connections. Previous reports using this type of cultures have also shown effects of NMDAR antagonists on the structure of hippocampal pyramidal neurons: treatment with MK-801 for 7 days did not alter pyramidal spine density on CA1, but produced an increase in the number of filopodia-like processes, which resemble the immature spines observed during synaptogenesis in the developing hippocampus (McKinney et al., 1999a). Only a piece of previous work in primary cultures has shown that the chronic exposure to ketamine produces a retraction of the dendritic arbor of interneurons in cell cultures (Vutskits et al., 2007). Although the phenotype of these interneurons was not analyzed, these results are similar to those in the present study, which show a reduction in the density of dendritic spines and thus in the area susceptible for stablishing synaptic contacts.

Even though axonal remodeling has not been as broadly studied as that involving dendrites, some studies have also highlighted the effects of NMDAR antagonists on axonal structure. Only 3 days of MK-801 treatment are enough to produce axonal sprouting in the hippocampal Schaffer collaterals *in vitro* (McKinney et al., 1999b). We have observed a similar effect in adult animals on the axonal *boutons* of somatostatin-expressing interneurons. These collaterals in the *stratum lacunosum moleculare* are most likely emerging from O-LM cells, since they are the only somatostatin-expressing interneurons innervating this area (Freund and Buzsáki, 1996). Whether a similar effect on Schaffer collaterals occurs *in vivo*, still remains to be explored. However, it is interesting to note that a recent study has found that O-LM cells facilitate the transmission of intrahippocampal information through this input from CA3 and reduce the influence of the entorhinal afferents (Leão et al., 2012); the axonal terminal field of O-LM interneurons is located precisely in the same location of the entorhinal afferents to CA1 pyramidal neurons (Freund and Buzsáki, 1996).

Treatments with MK-801 have been extensively used to model the etiopathology of schizophrenia in rodents, because NMDAR antagonists can reproduce some symptoms of schizophrenia in normal individuals. In fact, there is a NMDA hypothesis of schizophrenia, which poses that alterations in the NMDARs, particularly during development, are involved in the etiopathology of this disorder (Adell et al., 2012; Lim et al., 2012). Since these receptors are expressed in certain interneuron populations, including somatostatin-expressing cells (Nyíri et al., 2003), it has already been speculated that a NMDAR hypofunction during development would inhibit interneurons, thus producing an overexcitation of the network. Different studies point to this hypofunction as a factor contributing to alterations of fast-spiking neurons in schizophrenia (Coyle, 2006; Lisman et al., 2008), but alterations of these receptors in other interneuronal populations could also result in an abnormal functioning or connectivity. Based in our present results and previous ones describing the structural plasticity of dendrite-targeting interneurons (Guirado et al., 2014a,b; Gilabert-Juan et al., 2017), it is reasonable to speculate that a hypofunction of NMDAR might result in changes in the postsynaptic (spines) and presynaptic (EPB) elements. Whether these changes occur in schizophrenic patients still remains to be determined. However, interestingly, in a recently developed double hit model of schizophrenia using the same strain of this study, we have observed a reduction of spine density in EGFP-labeled interneurons in the mPFC (Castillo-Gómez et al., 2017).

In conclusion, our results set a promising starting point to understand the dynamics of interneuronal spines, how this structural plasticity can be regulated by NMDARs and what are the mechanisms underlying it. With a better understanding of the structural remodeling of interneurons we may predict its impact on local neuronal networks and, in time, understand better the neurobiology of complex psychiatric disorders, such as schizophrenia or major depression, in which both NMDARs and inhibitory networks are affected.

## Author Contributions

EC-G, RG, JN and MP-R designed the study. MP-R, EC-G and RG performed the experiments. MP-R analyzed the data prepared the figures. MP-R, EC-G, JMB-I, CC, EV and JN wrote the manuscript.

## Conflict of Interest Statement

The authors declare that the research was conducted in the absence of any commercial or financial relationships that could be construed as a potential conflict of interest.
